# Cleavage of AUF1 by Coxsackievirus B Affects DDX5 Regulatory on Viral Replication through iTRAQ Proteomics Analysis

**DOI:** 10.1155/2022/8610467

**Published:** 2022-10-06

**Authors:** Tianying Wang, Yujing Qin, Jiansheng Chen, Shuang Chen, Jingqi Wu, Xuepin Chen, Xiaomeng Chen, Shaofei Zhou, Jianhong Zou, Jun Guan

**Affiliations:** ^1^Clinical Research Center, Qingdao Municipal Hospital, Qingdao, China; ^2^Department of Gastroenterology, Heilongjiang Provincial Hospital, Harbin, China; ^3^Department of Gastroenterology, Heilongjiang Provincial Hospital, Harbin Institute of Technology, Harbin, China; ^4^Department of General Surgery, Qingdao Municipal Hospital, Qingdao, China; ^5^Department of Clinical laboratory, Qingdao Municipal Hospital, Qingdao, China; ^6^Department of Microbiology, Harbin Medical University, Harbin, China; ^7^Department of Cardiology, Qingdao Municipal Hospital, China; ^8^Center for Disease Prevention and Control of Qingdao Shibei District, Qingdao, China

## Abstract

Coxsackievirus B (CVB) 3C protease (3C^pro^) plays a specific cleavage role on AU-rich binding factor (AUF1, also called hnRNP D), which consequently disputes the regulation of AUF1 on downstream molecules. In our study, the iTRAQ approach was first used to quantify the differentially expressed cellular proteins in AUF1-overexpressing HeLa cells, which provides straightforward insight into the role of AUF1 during viral infection. A total of 1,290 differentially expressed proteins (DEPs), including 882 upregulated and 408 downregulated proteins, were identified. The DEPs are involved in a variety of cellular processes via GO terms, protein–protein interactions, and a series of further bioinformatics analyses. Among the DEPs, some demonstrated important roles in cellular metabolism. In particular, DDX5 was further verified to be negatively regulated by AUF1 and increased in CVB-infected cells, which in turn promoted CVB replication. These findings provide potential novel ideas for exploring new antiviral therapy targets.

## 1. Introduction

The cellular life cycle of picornaviruses is dependent on the modification of multiple cellular processes and the repurposing of host proteins for progeny virion generation [1]. Although the replication of picornaviruses, including enteroviruses, is carried out strictly in the host cytoplasm, viral infection can induce the cytoplasmic translocation of specific nuclear proteins [2]. Picornavirus-encoded proteins may cause the shutdown of multiple host cell functions, including cellular transcription and cap-dependent translation, as well as the disruption of innate immune signaling and nucleocytoplasmic transport [3]. These cellular processes are accompanied mainly by the cleavage of key host cellular proteins by virus-encoded proteinases. For picornaviruses, including human rhinoviruses (HRV), poliovirus, and coxsackievirus group B (CVB), the proteinases encoded by them include 2A protease (2A^pro^) and 3CD/3C protease (3CD^pro^/3C^pro^). Picornavirus 3C^pro^ has several cellular protein targets, as reported, and their cleavage could directly facilitate the viral replication cycle or disrupt host cellular processes to protect the virus. For instance, cleavage of NF-*κ*B disrupts the host cellular innate immune response [3, 4].

AU-rich binding factor 1 (AUF1), also known as hnRNP D, has recently been reported to be cleaved by poliovirus and CVB [2, 5]. AUF1 has characteristics similar to those of an hnRNP, including nucleocytoplasmic shuttling properties and multiple isoforms [6]. AUF1 consists of four isoforms (p45, p42, p40, and p37); of all isoforms, p45 is the largest. As a result of cleavage, picornaviruses can either inhibit/alter the function of AUF1 or exploit the function of their cleavage products and the downstream-regulated proteins [7]. In addition, AUF1 is one of at least six RNA-binding proteins involved in AU-rich element (ARE)-mediated mRNA decay (AMD), such as zinc finger proteins, tristetraprolin (TTP) [8], and the DEAD-BOX protein family (DDX family) [9]. During CVB infection, previous data showed that AUF1 was not only recruited into stress granules (SGs) [10] that formed in host cells under unfavorable stimulation, such as virus infection [11], and affected mRNA localization, translation and degradation, as well as signaling pathways and antiviral responses [12]; AUF1 was also reported to negatively regulate viral infection by inhibiting viral translation [3]. However, picornaviruses could react to AUF1 by its 3C^pro^ cleavage [2]. To comprehensively illustrate the function of AUF1 and potential networks under viral infection, we performed proteomics analysis in EGFP-AUF1 p45-overexpressing HeLa cells to reveal the global regulatory function of AUF1 on the proteomic profile. The study could provide us with a broader perspective from which to screen potential therapeutic targets for CVB infection.

The DEAD-box family of putative ATP-dependent RNA helicases (DDXs) contains a series of proteins involved in the regulation of a large number of cellular RNA metabolic processes [13], including replication, DNA repair, RNA stability, translation initiation, and transcription. They have the ability to remodel RNA–RNA or RNA–protein complexes.

Picornaviruses, such as CVB, have relatively small genomes that encode a limited number of viral proteins that may impede host cellular components and pathways to facilitate viral replication [14]. In particular, DDXs were reported to be involved in a series of cellular processes of RNA viruses. DDX3, together with DDX1, has been found to promote the nuclear export of viral mRNAs in human immunodeficiency virus-1 (HIV-1) [15] and be required for genome replication in hepatitis C virus (HCV) [16]. Moreover, the DDXs also participate in the later stages of viral infection. For instance, DDX24 and DDX56 are involved in HIV-1 and West Nile viral particle assembly, respectively [17]. Notably, DDX5 has been identified as a double-edged sword in various types of viruses. It has been recently documented to play a promoting effect on viral replication, such as severe acute respiratory syndrome (SARS) coronavirus (CoV), HIV, HCV, and other RNA viruses, while it has an inhibitory role in DNA viral replication, including hepatitis B virus (HBV) and myxoma virus (MYXV) [18].

These results imply that during CVB infection, cleavage of AUF1 by CVB 3C^pro^ may influence DDX proteins, which may be beneficial for viral replication. In the present study, TMT technology was used to analyze proteome changes in response to AUF1 overexpression compared with the control cells. The DEPs were analyzed to achieve a better understanding of the effects of AUF1. GO and KEGG enrichment analyses of DEPs were applied, and the protein–protein interaction (PPI) networks and clusters of the selected DEPs were also investigated. The aim of this study was to identify the key proteins and pathways in AUF1-overexpressing host cells using a bioinformatics approach and biological assays. We aimed to identify the potential mechanisms and new host cellular molecules that may be novel potential diagnostic and therapeutic biomarkers under CVB infection. We anticipated that AUF1 overexpression-induced proteomics profiling changes could provide further insight into virus–host interactions at the molecular level.

## 2. Materials and Methods

### 2.1. Cell Culture and Transfections

Human HeLa cells were cultured in high-glucose Dulbecco's modified essential medium (Gibco, Cat No. 11965092, USA) supplemented with 10% fetal bovine serum (CellMax, Cat No. SA211.02, Beijing, China) and antibiotics (Gibco, Cat No. 15640055, USA). Cells were plated at 80-90% confluence and transfected with EGFP-AUF1 fusion plasmids (isoforms p45) or siRNAs (Cell Signaling Technology, Cat No. 12763, USA). Transfections were performed using Lipofectamine 2000 (Invitrogen, Cat No. 11668019, USA). Cells were then collected for analysis 48 h after transfection, and total cell lysis was collected for further analysis.

### 2.2. Sample Preparation and Trypsin Digestion

Cells were harvested with RIPA buffer (Thermo, Cat No. 89901, USA), and the concentration was detected with a BCA kit (Beyotime, Cat No. P00125, Beijing, China). Further proteomic LC–MS/MS experiments were performed by Jingjie PTM Biolab Co., Ltd. (Hangzhou, China). Briefly, all lysis was performed with trypsin digestion. Each sample was reduced with 5 mM DTT at 56°C for 30 min and alkylated with 11 mM iodoacetamide (IAA, Sigma) for 15 min at room temperature in darkness. Then, trypsin was added at a ratio of 1 : 50 (trypsin to protein) to perform the first overnight digestion, and a ratio of 1 : 100 was used for a second 4-h digestion to improve the digestion efficiency [19].

### 2.3. Stable Isotope Dimethyl Labeling

After trypsin digestion, the peptide was desalted by Strata X C18 (Phenomenex) and vacuum dried. The peptide was dissolved in 0.5 M TEAB and differentially isotope labeled in parallel in different tubes. The samples were mixed briefly, and a 1-mg mixture of samples was dried by vacuum centrifugation.

### 2.4. HPLC Fractionation

The labeled peptides were fractionated into 80 fractions using an Agilent 1260 zorbax extend - C18 column (4.6 mm ×250 mm, Agilent Technologies, Santa Clara, CA, USA). Then, the peptides in 80 tubes were combined into 18 fractions that were used in the subsequent LC–MS/MS analysis of the whole-cell proteome.

### 2.5. LC–MS/MS Analysis

The enriched peptides and peptides digested from the whole-cell proteome were dissolved in 0.1% formic acid. Peptides were loaded onto an EASY-nLC 1000 UPLC system (Thermo Scientific™ Q Exactive™ Plus, MA, USA) and separated on an Acclaim PepMap RSLC reverse-phase analytical column (Thermo Fisher Scientific, Waltham, MA, USA). For whole-cell proteome analysis, the gradient was 7% to 20% solvent B (0.1% formic acid in 98% acetonitrile) over 24 min, followed by 20% to 35% solvent B for 8 min, increased to 80% over 5 min, and finally held at 80% solvent B with a flow rate of 300 nl/min for the last 3 min.

The peptides were ionized by a nanospray ionization source, followed by MS/MS in a Q Exactive™ Plus tandem mass spectrometer (Thermo Fisher Scientific, Waltham, MA, USA). Intact peptides were detected in an Orbitrap at a resolution of 70,000. The mass range for the MS scans was 350 to 1800 m/z. A data-dependent analyzer (DDA) was adopted that alternated between one MS scan followed by 20 MS/MS scans. To optimize the mass of the secondary MS, the ions must accumulate over 5E3 to perform the secondary analysis. Automatic gain control was used to prevent overfilling of the Orbitrap when 5E4 ions were accumulated for generation of MS/MS spectra. The electrospray voltage was set at 2.0 kV. Dynamic exclusion was set as 15 s for the whole-cell proteome to reduce the repeat identification of peptides.

### 2.6. Database Search

The MS/MS data were processed by Mascot 2.3 software. Tandem mass spectra were searched for Homo sapiens. Meanwhile, the target-decoy search strategy was employed to eliminate the false-discovery rate (FDR). Trypsin/P was specified as a cleavage enzyme allowing up to 2 missing cleavages. The mass error was set to 10 ppm for the first search and 0.02 Da for secondary ions. Cys-Carbamidomethylation was specified as fixed modification. For quantification, the FDR thresholds for peptides were specified at 1%. The minimum peptide length was set at 7. The mass spectrometry proteomics data have been deposited to the ProteomeXchange Consortium via the PRIDE [20] partner repository with the dataset identifier PXD021094.

### 2.7. Data Preprocessing

There were three technical replicates in the proteomic examination, and the repeatability test was carried out for each protein. The *P* value was calculated, and *P* < 0.05 was considered to be repeatable for the three tests of the protein.

### 2.8. Differentially Expressed Protein (DEP) Screening and Enrichment Analysis

The R package “Limma” was used to screen the differentially expressed proteins between the AUF1-overexpressing and control groups. Threshold FDR<0.05 and |log2FC| > 1.2 criteria were set for the DEP definition. To explore the majority of the differentially expressed proteins functions and their counterpart biological pathways, we performed GO and KEGG enrichment analyses to identify functions and pathways using DAVID database v6.8 (https://david.ncifcrf.gov/). The top enriched terms (ranked by enriched score) in the enrichment analysis were considered strongly enriched terms.

### 2.9. Protein–Protein Interaction (PPI) Network Analysis

The protein–protein interaction (PPI) dataset of DEGs was obtained from String (http://string-db.org). The degree of a node was counted by the edges that linked it with others. The width of edges indicated the combined score estimated by the database. Cytoscape v3.6.1 was used to analyze the PPI network topological structure, and Centiscape2.2 and MCODE APP were used to measure node degrees and screen gene clusters. Hub nodes were identified using the cutoff of degree ≥50, betweenness ≥10000, and closeness ≥3.25E-4. The threshold was set to at least 3 hub nodes in the cluster identified by MCODE.

### 2.10. RNA Sample Preparation for RT–qPCR Validation

RNA samples were extracted from EGFP-AUF1 p45-overexpressing HeLa and control cells. Cells were lysed with TRIzol (Invitrogen, Cat No. 15596026, USA), followed by isopropanol precipitation and removal of salts with 75% v/v ethanol/H_2_O. We further removed traces of genomic DNA from the samples by gEraser treatment and performed reverse transcription with the PrimeScript™ RT reagent kit with gDNA Eraser (Perfect Real Time) (Takara, RR047A, Beijing, China). To validate gene-specific total mRNA levels, we used TB Green Premix Ex Taq™ II (Tli RNaseH Plus) reagent (Takara, Cat No. RR820 L, Beijing, China). We collected the melting curves on a StepOne Plus Real-Time PCR System (Applied Biosystems 7500, USA) and analyzed the data on the instrument's software version 2.2.2.

### 2.11. Western Blots

Cells were transfected with EGFP-AUF1 p45 24 h before harvest. Total cell lysates were prepared with RIPA lysis buffer and detected by SDS–PAGE. Then, the proteins were transferred to a 0.22-*μ*m PVDF membrane and detected with a specific primary antibody. DDX5 antibody (Abcam, ab126730, USA) was used, and *β*-tubulin (Origene, TA310155, Beijing, China) was used as a loading control. Gray values of bands were calculated by ImageJ software.

### 2.12. Virus and Cells

The CVB3 woodruff strain was used in our study, which was preserved by our lab. HeLa cells were infected with virus (MOI = 10, 6 h).

## 3. Results

### 3.1. Identification of Differentially Expressed Proteins

EGFP-AUF1-overexpressing and nontreated control HeLa cells were analyzed, and lysis was collected 24-h post-transfection. Using iTRAQ-based quantitative proteomic analyses, based on the differential analysis, using the *P* < 0.05 and |log2FC| > 1.2 criteria, a total of 1,290 DEPs were identified, consisting of 882 upregulated and 408 downregulated DEPs in EGFP-AUF1-overexpressing HeLa cells compared with nontreated control cells.

### 3.2. Functional Classification of Enriched DEPs by GO Term

**Figure 1:**
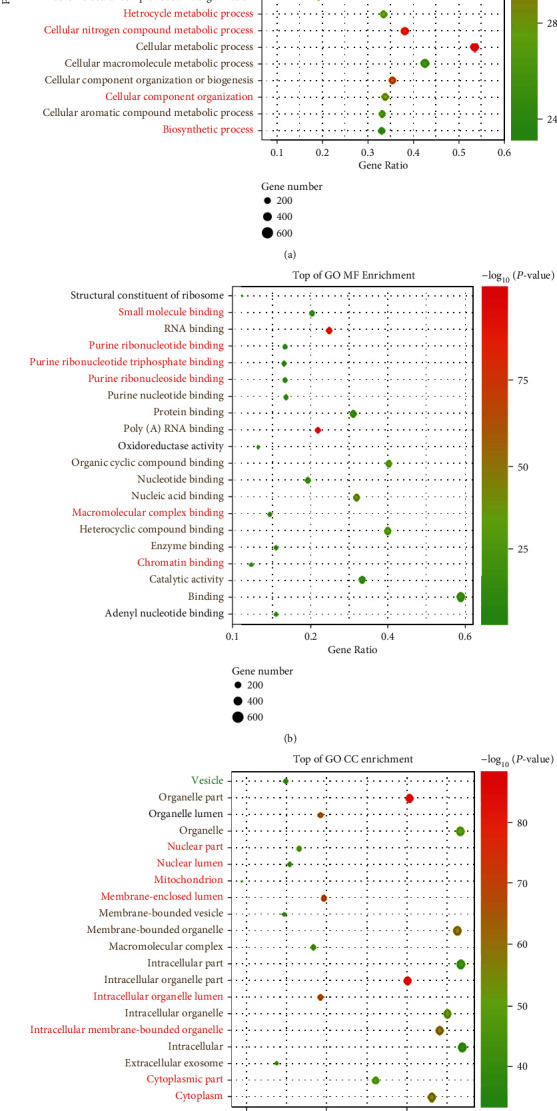
GO term enrichment analyses of DEPs in the AUF1-overexpressing group vs. the control group. GO term analyses of proteomics that were cataloged as (a) biological process (BP); (b) molecular function (MF); and (c) cellular component (CC). The top 20 significant GO term enrichment results are shown (*P* < 0.05). Terms enriched in both upregulated and downregulated protein sets are marked with a gradient color (red to green); terms also enriched in the upregulated protein set are marked in red, while green indicates the downregulated protein set. All of the BP, MF, and CC terms were ranked in terms of enrichment of the differentially expressed proteins, and the top 20 are presented here.

To perform further investigation of the putative functions of the identified DEPs, the DEPs were uploaded to the GO and KEGG databases for cellular component, molecular function and biological process categories (CC, MF, and BP), and pathway enrichments. The R package ggplot2 was used for GO result display (Figure 1). A total of 195 terms were enriched in the CC category, 74 terms in MF, and 505 terms in BP. A term was defined as significantly enriched when it had a *P* value <0.05. For GO analysis, all DEPs were markedly enriched in BPs, including multiple metabolic processes and some biosynthetic processes (Figure 1(a)). Based on the “-log_10_ (*Pvalue*),” all of the GO terms in the three categories were significantly enriched. For molecular function (MF), the DEPs were significantly enriched in nucleotide, RNA, and enzyme binding (Figure 1(b)), and in GO cellular component (CC) analysis (Figure 1(c)), DEPs were significantly enriched in organelles, including intracellular organelles, organelle parts, and membrane-bounded organelles. GO term analyses were also performed on upregulated proteins or downregulated proteins separately. To provide authors an intuitive result, we highlighted the terms that were enriched in the upregulated and downregulated protein set in red and green, respectively. Terms enriched both in upregulated and downregulated protein sets are shown in gradient color. For BP, most terms overlaid with results in the upregulated protein set (Figure 1(a), red and gradient color terms), indicating that upregulated proteins contributed more. The amounts of molecules involved in the RNA post-transcriptional modification process were included, such as CPSF, CSTF, DDX, and EIF. AUF1 controls mRNA stability [21], which regulates multiple cellular processes, including transcriptional activation and pre-mRNA processing in the nucleus, while binding to several mRNAs, such as c-myc, c-fos, and cyclin D1.

However, GO term BP enrichment for the downregulated protein set is shown in Figure S1. We found that the significantly downregulated proteins were involved in BPs, including multiple catabolic processes and, interestingly, some virus-related processes. As our previous research noted, during viral infection (Coxsackievirus Group B), AUF1 played a role in viral mRNA stability and the inflammatory response [2, 22]. Therefore, the downstream target of AUF1 involved in viral infection received more attention. Among DEPs in this subcategory, ANXA3, a member of the annexin family of calcium-dependent, phospholipid-binding proteins [23], has been reported to promote virion maturation and function as a host factor required for efficient HCV particle production. Metabolism mainly consists of anabolism and catabolism. Thus, we marked the corresponding terms in Figure 1(a), in which catabolic processes were involved in metabolic processes (Figure 1(a), green and gradient color). In summary, GO term enrichment analyses further explained that metabolism, biosynthesis, organelle, and RNA or protein binding should be tightly regulated.

### 3.3. Functional Classification of Enriched DEPs by KEGG

**Figure 2:**
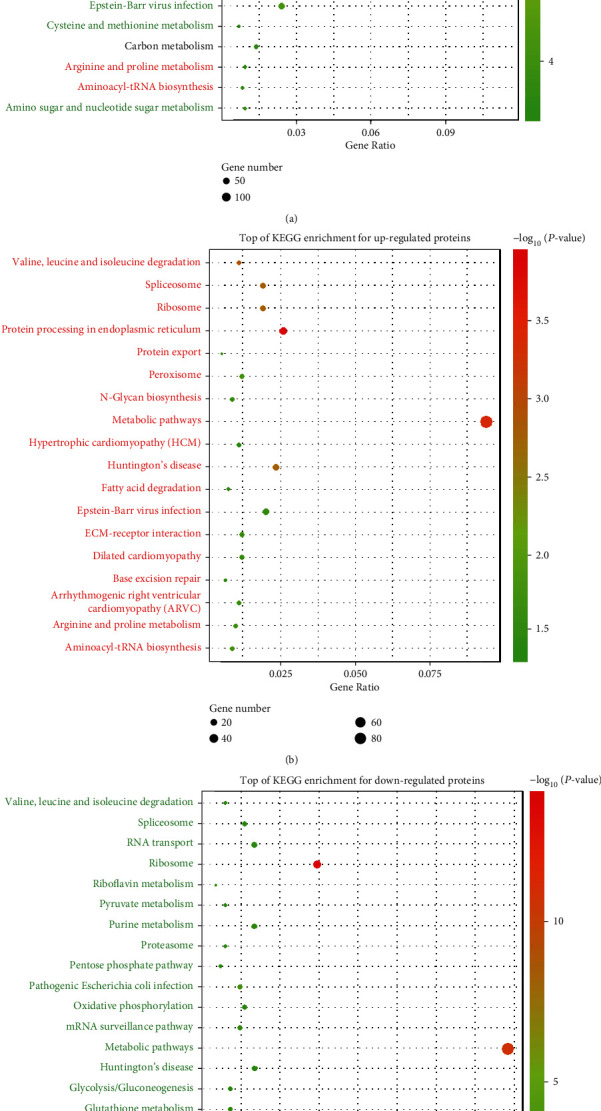
KEGG enrichment analyses of DEPs associated with the AUF1 overexpression group vs. the control group. KEGG analyses of proteomics that were cataloged as (a) top pathways enriched with all the DEPs. Upregulated pathways are marked in red, while downregulated pathways are marked in green. Those enriched in both up- and downregulated pathways are marked with a gradient color (red to green); (b) top upregulated pathways enriched by KEGG are listed; (c) Top downregulated pathways enriched by KEGG are listed. Each dot represents the pathways, the color represents the *P* value, and the size of the dot represents the number of DEPs enrolled. The proportion of genes is represented by the *x*-axis.

To further investigate the signaling pathways enriched in DEPs, a KEGG pathway analysis was performed using a standard of FDR <0.05. As shown in Figure 2, the top 20 pathways are shown in a bubble plot (Figure 2(a)). The pathway analysis results demonstrated that the metabolism pathway was significantly enriched (gene ratio =0.114) and associated with AUF1 overexpression (FDR<0.001). The metabolism pathway was also found in both the upregulated protein set and downregulated protein set KEGG enrichment analyses (Figures 2(b) and 2(c)).

### 3.4. Protein-Protein Interaction (PPI) Analysis of the DEPs

**Figure 3:**
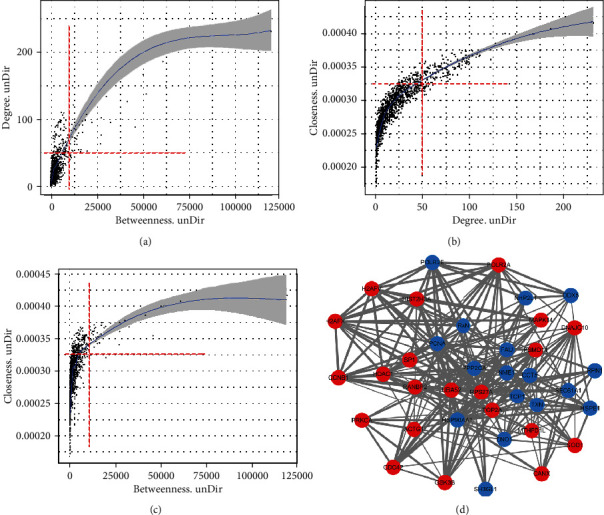
Protein–protein interaction network (STRING) obtained from analyzing the products of DEGs. (a, b, and c) The nodes were analyzed every two factors. (d) Thirty-eight DEPs were mapped in the PPI networks. Using Cytoscape 3.6.1, DEPs were mapped in PPI networks. The thresholds of the three parameters were set as follows: degree =50, betweenness =10000, closeness =3.25E-4, and 95% confidence level (STRING score) =0.4. The thickness of the lines indicates the number of interactions that are detailed in STRING (http://string-db.org).

The 1,290 DEPs identified in the current study were submitted to Cytoscape 3.6.1 for further PPI network analysis (Figure 3). Of the 1290 identified DEPs, 38 DEPs were mapped in PPI networks. Using Cytoscape 3.6.1, we set the threshold of three parameters (degree =50, betweenness =10000, and closeness =3.25E-4) and 95% confidence level (STRING score =0.4). The nodes had been analyzed on every two factors (Figures 3(a)–3(c)). Those nodes had significantly larger values were selected and were taken the intersection. Through the approach, 38 hub notes had been selected (Figure 3(d)). These hub nodes not only connected with multiple other nodes, but also had a small value of the shortest path linked to other nodes. And more frequently worked as intermediate bridges between the shortest path of other nodes. All indicated that these hub nodes (Figure 3(d), blue and red dots represented downregulation and upregulation, respectively) played a vital role among the whole PPIs network. The thicker lines indicated that the molecules played stronger and more important effects among the interactions, including DDX5, POLR2A, and Uba52. They were most probably affected by the change of AUF1 expression and even might be the direct regulatory target of AUF1 or other nucleic-binding proteins. For instant, it has been reported that human Uba52 gene encode Ub fused to the ribosomal proteins (RPs), like RPS27a (also a hub node in our study). RPs have a high proportion of motif that is common to some nucleic acid-binding proteins 22. Hubs in the network revealed the potential key role of the molecules and exhibited a complex relationship with the other proteins, such as DDX5, POLR2A, MAPK14, and PPP2CA.

### 3.5. Protein–Protein Interaction (PPI) Analysis of the DEPs

The 1,290 DEPs identified in the current study were submitted to Cytoscape 3.6.1 for further PPI network analysis (Figure 3). Of the 1,290 identified DEPs, 38 DEPs were mapped in the PPI networks. Using Cytoscape 3.6.1, we set the threshold of three parameters (degree =50, betweenness =10000, and closeness =3.25E-4) and 95% confidence level (STRING score =0.4). The nodes were analyzed for every two factors (Figures 3(a)–3(c)). Those nodes with significantly larger values were selected and taken as the intersection. Through this approach, 38 hub nodes were selected (Figure 3(d)). These hub nodes not only connected with multiple other nodes but also had a small value of the shortest path linked to other nodes. In addition, they more frequently functioned as intermediate bridges between the shortest paths of other nodes. All indicated that these hub nodes (Figure 3(d), blue and red dots represent downregulation and upregulation, respectively) played a vital role among the entire PPI network. The thicker lines indicate that the molecules played a stronger and more important role in the interactions, including DDX5, POLR2A, and Uba52. They were most likely affected by the change in AUF1 expression and may even be direct regulatory targets of AUF1 or other nucleic-binding proteins. For instance, it has been reported that the human Uba52 gene encodes Ub fused to ribosomal proteins (RPs), such as RPS27a (also a hub node in our study). RPs have a high proportion of motifs that are common to some nucleic acid-binding proteins [24]. Hubs in the network revealed the potential key role of the molecules and exhibited a complex relationship with other proteins, such as DDX5, POLR2A, MAPK14, and PPP2CA.

**Figure 4:**
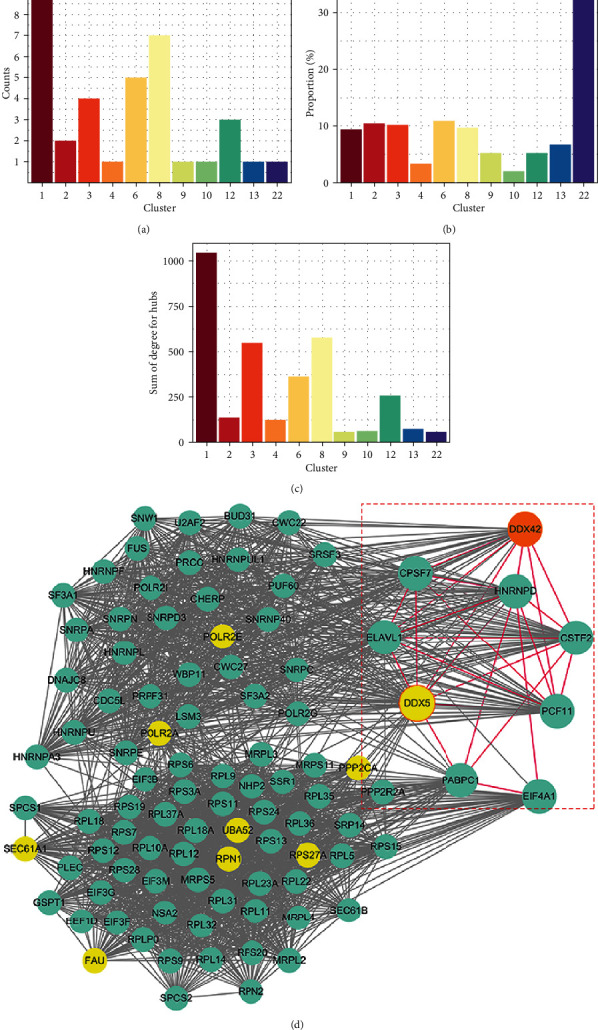
The clusters in the network (*k* = 3). We numbered the clusters from 1 to 29 in descending order according to the MCODE score analyzed with the Cytoscape plugin. (a) Clusters that had more hub nodes were selected. (b) Clusters that had a higher proportion of all genes were selected. (c) Clusters were selected that had a larger value for the sum of parameter “degrees.” (d) Cluster 1, which contained the most hubs, was selected, and hubs are highlighted in yellow background. The red dashed box shows the hub nodes connected with AUF1 directly and mainly played roles in RNA regulation. Red lines indicate the interactions between the hubs.

**Figure 5:**
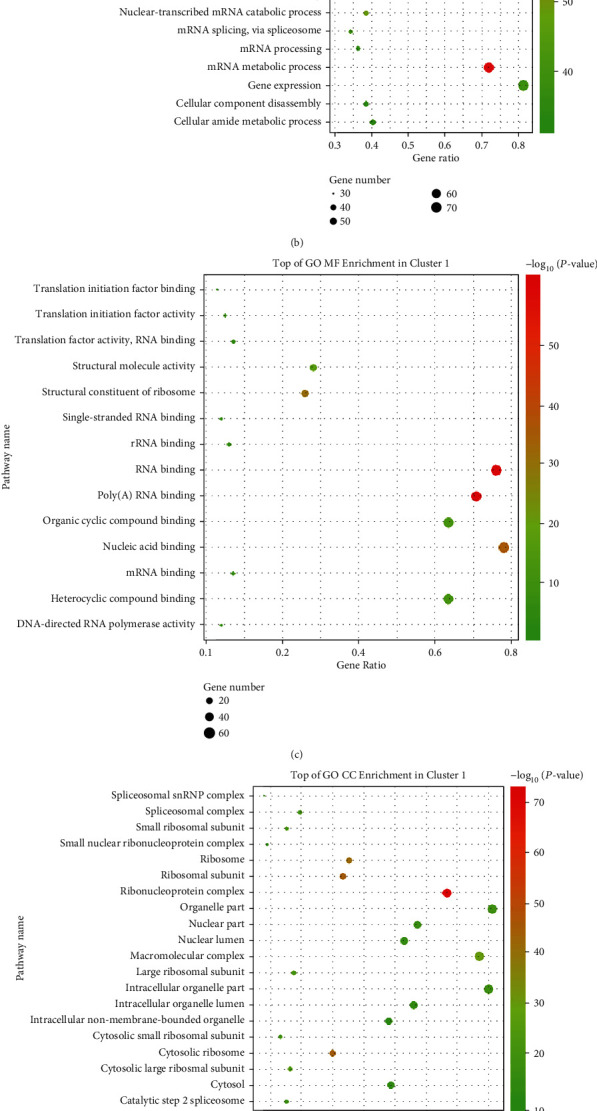
GO term and KEGG enrichment in cluster 1 and the DDX-centric subnetwork. (a) DDX-centric subnetwork module. (b) Top of GO BP enrichment in cluster 1. (c) Top of GO MF enrichment in cluster 1. (d) Top of GO CC enrichment in cluster 1. (e). KEGG enrichment in cluster 1. Each dot represents the pathways, the color represents the *P* value, and the size of the dot represents the number of DEPs enrolled. The proportion of genes is represented by the *x*-axis.

Furthermore, the Cytoscape plugin and MCODE analysis revealed that there were 29 clusters in the network (*k* = 3), which were the sets composed of the proteins tightly associated with each other and probably involved in the same cellular process. The clusters were numbered from 1 to 29 in descending order according to the MCODE score. We focused on the clusters containing hub nodes that may be even more important in multiple processes (clusters 1, 2, 3, 4, 6, 8, 9, 10, 11, 12, and 22), especially those that had more hub nodes (Figure 4(a)), those in which hub nodes accounted for a higher proportion of all genes (Figure 4(b)), or those that had a larger value for the sum of parameter “degrees” (Figure 4(c)). Based on the above consideration, clusters containing more hub nodes screened clusters 1, 3, 6, and 8 as the best clusters, especially cluster 1, which contained the most hubs (Figure 4(d), hubs highlighted in yellow background) compared with the other three clusters, while cluster 22 was ruled out as it only contained a total of three nodes, which caused the significantly high hub proportion in that cluster (Figure 4(b)). In cluster 1, compared with other hub-centric subnetworks (Figure S2), we focused on the DDX5-centric subnetwork, which interacted with a large number of proteins (Figure 5(a)), including many RNA-binding proteins involved in processes from RNA transcription to translation, polyadenylation, splicing, and metabolism, such as PABPC1, PCF11, and hnRNP D (AUF1).

Moreover, DEPs in cluster 1 were introduced into GO and KEGG databases for enrichment analyses (Figures 5(b)–5(e)) to further explore the biological functions and pathways in which they were involved. For GO BP analysis, DEPs in cluster 1 were markedly enriched in BPs, including multiple metabolic (or catabolic) processes; interestingly, some were enriched in viral transcription- or gene expression-related processes (Figure 5(b)). Based on the “-log10 (*P* value),” all of the GO terms in the three categories were significantly enriched. Regarding molecular function (MF), the DEPs were significantly enriched in binding functions, including RNA, poly(A) RNA and nucleic acid binding (Figure 5(c)), and in the GO cellular component (CC) analysis (Figure 5(d)), DEPs were significantly enriched in many complex components, including ribonucleoproteins and macromolecule complexes. Examination of the details of the parameters of DEPs in cluster 1 revealed that DDX5, POLR2E, and POLR2A were the top three hub nodes that had the highest MCODE scores (39). For KEGG enrichment, ribosome and spliceosome predominated significantly among the pathways (Figure 5(e)).

### 3.6. Negative Regulatory Role of AUF1 on DDX5 mRNA

Hub node DDX5 has been reported to participate in multiple aspects of RNA metabolism, ranging from transcription to translation, RNA decay, and RNA processing [18]. We investigated the details of the DDX5-centric subnetwork diagram (Figure 5(a)). DDX5 not only interacted with other important hubs (POLR2A and POLR2E) (Figure 5(a), red dots) but also contacted some other members of the DDX family, such as DDX42, DDX24, and DDX18 (Figure 5(a), orange dots). Furthermore, it showed a direct interaction with hnRNP D (AUF1, highlighted by red circle), which implied that DDX5 may be significantly regulated or affected by the expression change of AUF1.

**Figure 6:**
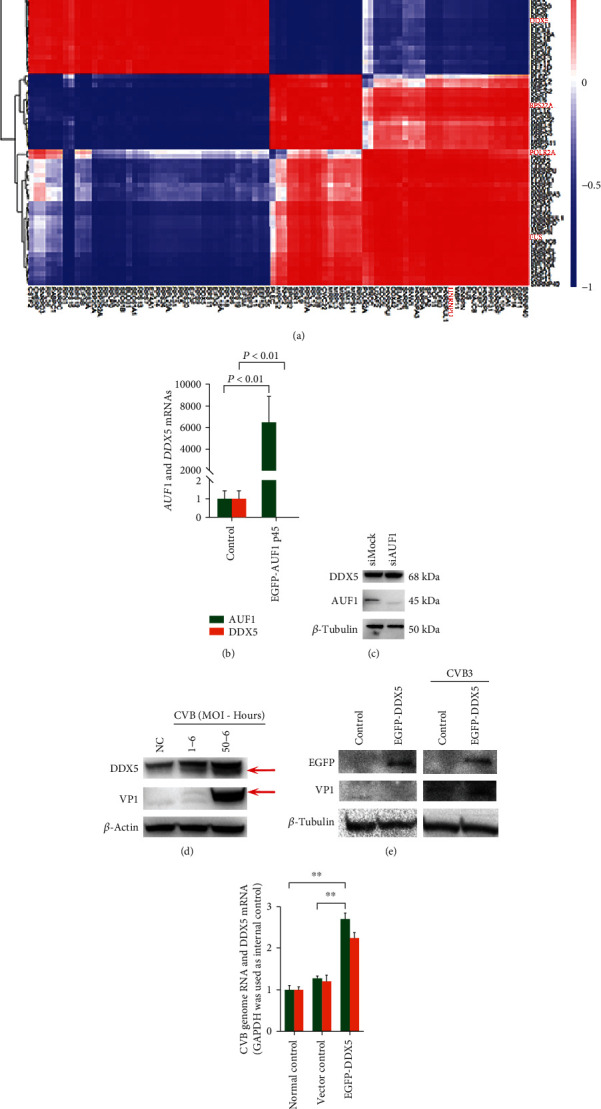
Correlations and validation of AUF1 regulation of DDX5 w/w.o viral infection. (a) Heatmap of correlations between proteins in cluster 1 and analysis of bidirectional hierarchical clustering. (b) The RT–qPCR assay was used to examine the DDX5 mRNA expression level with or without AUF1 overexpression (*t* test, *P* < 0.01). GAPDH was used as an internal control. (c) DDX5 protein expression levels were detected with or without silencing AUF1 with siRNA by Western blot analysis. *β*-Tubulin was used as a loading control. (d) DDX5 protein levels were detected under the condition that AUF1 was cleaved during CVB infection at different MOIs and infection hours. VP1 was used for viral detection, and *β*-tubulin was used as a loading control. (e) CVB VP1 was examined in HeLa cells with or without EGFP-DDX5 overexpression by Western blot analysis. (f) CVB genome RNA expression levels in HeLa cells with or without EGFP-DDX5 overexpression were detected by RT-qPCR.

To verify this hypothesis, we first analyzed the correlations among proteins in cluster 1 based on expression spectrum data, which may provide more information on the relationship of DDX5 and others, especially hnRNP D. The correlation data are shown in a heat map (Figure 6(a)). Hub nodes are highlighted in red characters. DDX5 was significantly negatively correlated with hnRNP D (Pearson's correlation coefficient = -0.638, *P* value = 0.0643). Hierarchical cluster analysis could significantly divide the proteins into two groups, which were upregulated and downregulated under AUF1 overexpression conditions. Principal KEGG enrichment can be significantly divided into two clusters and analyzed (Supplementary Table 1, the upregulated group has an orange background, and the downregulated group has a green background). This result indicated that AUF1 overexpression treatment predominantly induced the differential expression of proteins involved in the spliceosome and ribosome, the key steps of gene expression.

Based on these analyses, further biological effects of AUF1 on DDX5 expression were investigated in HeLa cells. We transfected HeLa cells with EGFP-AUF1 p45 and control vector for 24 h, and *DDX5* mRNA expression was then examined by the RT–qPCR assay. Compared with the control group, *DDX5* mRNA was significantly decreased in the EGFP-AUF1 p45-overexpressing group (Figure 6(b), *P* < 0.01).

### 3.7. CVB Replication Was Promoted by DDX5 Overexpression Associated with AUF1 Expression

**Figure 7:**
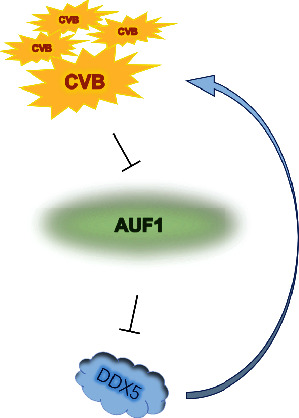
Schematic diagram of the interaction between DDX5, AUF1, and CVB. AUF1 is cleaved under CVB infection; thus, the inhibitory effect of AUF1 on DDX5 was suppressed. DDX5 further increases and improves CVB replication.

Based on our previous data, CVB could cleave AUF1 by its 3C protease [22]. To elucidate the consequent effect of DDX5 regulation by AUF1 during CVB infection and its potential role in viral replication, we first knocked down AUF1 expression in HeLa cells with siRNA. Compared with the control group, DDX5 was improved with AUF1 expression silencing (Figure 6(c)). This result indicated that AUF1 played a negative regulatory role on DDX5 mRNA and protein (Figures 6(b) and 6(c)), which was in accordance with the PPI and correlation analysis results. Then, we infected cells with CVB at different MOIs and detected the DDX5 expression level (Figure 6(d)). There was an obvious increase in DDX5 under CVB infection at an MOI = 50 for 6 hours. To illustrate the role of the DDX5 increase in CVB infection, we used EGFP-DDX5 to transfect HeLa cells, and the CVB replication level was then detected (Figure 6(e)). Compared with the control group without DDX5 overexpression, the VP1 expression that indicated the CVB replication level was significantly increased at the early time point (6 hours), and CVB genome RNA levels were also detected in the normal control, vector control, and EGFP-DDX5 overexpressed groups. Compared with the normal and vector control groups, EGFP-DDX5 overexpression induced an increase in CVB genomic RNA (Figure 6(f), *P* < 0.01 and *P* < 0.01, respectively). In conclusion, CVB cleaved AUF1 and consequently increased DDX5 expression, which in turn improved viral replication (Figure 7).

## 4. Discussion

The study presented above provides new mechanistic insights into CVB based on the host DDX increase, which is induced by CVB 3C^pro^ cleavage of AUF1. Proteomic profiling implies that AUF1 is tightly associated with multiple cellular pathways and plays a vitally important role during viral infection. It not only affects CVB genome RNA and other host mRNA stabilities but also interacts with many molecules involved in promoting viral replication or evasion of the host immune system. AUF1 was initially recognized as an mRNA decay protein [25]. Thereafter, it was found that AUF1 could bind to the CVB RNA 3'UTR and destabilize viral RNA in HeLa cells [1, 2]. Moreover, Bert L. Semler's group [1] recently demonstrated that AUF1 negatively regulated viral translation by acting as a negative ITAF during infection. In some sense, this may be the strategy that CVB used to defend the host immune system. From another point of view, the response of the host facing the cleavage of AUF1 may be very important for antiviral therapy. Here, we used EGFP-AUF1 p45-overexpressing HeLa cells to determine the proteomic profile changes compared with nontreated cells, which could make our work more targeted.

In our work, we performed GO and KEGG enrichment analyses of the DEPs between AUF1-overexpressing and control cells. Based on the three components of the GO term results, DEPs were mostly annotated in the cellular metabolism process and involved in binding functions. AUF1, as an RNA binding protein, has been reported to be involved in the degradation of oxidatively damaged RNA when cells are exposed to oxidative stress [26]. To further elucidate the DEP interactions between each other, protein interaction networks were exported for further topology analysis. Thirty-eight hub nodes were identified, and 29 clusters were screened. Based on set parameters (degree ≥50, betweenness ≥10000, and closeness ≥3.25E-4), we focused on the clusters containing the hub nodes, which were considered to be more important in terms of cellular functionality. Cluster 22 was excluded because it only has three nodes in the cluster. Due to the MCODE score, the other four clusters (clusters 1, 3, 6, and 8) were remarkably compact. Among the four clusters, cluster 1 showed the highest levels of hub node number and number of edges connected to other nodes. Regarding the ratio of hub nodes to all the nodes in the cluster, the value of cluster 1 was not the highest, but it was still comparable to the highest value of cluster 6. In addition, we also checked the GO and KEGG enrichments for hub nodes in cluster 6. Most hubs were annotated in the biological processes and pathways cross-linked with those in cluster 1 (data not shown), including GSK3*β*, DNAJC10, and nucleoside diphosphate kinase (NME1). NME1 played an antiviral role by regulating p53-mediated antiviral innate immunity in foot-and-mouth disease virus (FMDV-)-infected cells [27], and glycogen synthase kinase 3 *β* (GSK3 *β*) was also identified to participate in the GSK-3/ *β*-catenin axis and play a role in antiviral innate immunity [28]. Moreover, they influence numerous cellular activities, such as glucose metabolism and transcriptional regulation, which are also linked to the pathways enriched in cluster 1. For instance, DNAJC8, DDX5, and DDX42 were all included in cluster 1 and participated in metabolic processes (supplementary file: the BP in GO analysis was performed on Cluster 1, and detailed data are supplied). Interestingly, we found that molecules (such as POLR2 and RPL family proteins) in cluster 1 were also annotated in terms associated with viral transcription and translation processing. Based on the above considerations, cluster 1 was chosen for further investigation to illustrate the role of AUF1 in global proteomics and its potential effects during viral infection.

Nine hub nodes were enrolled in cluster 1 (Figure 4(d), yellow dots), and hub node-centric subnetworks were constructed (Figure S1). Two subunits of POLR2 identified as hub nodes were included in cluster 1. Substantial evidence has demonstrated that POLR2 is used to synthesize cellular mRNA and viral mRNAs. POLR2 is an enzyme complex composed of multiple subunits [29] and is primarily used to synthesize host and viral mRNAs. The hub nodes POLR2A and POLR2E were both contained in cluster 1 and predicted to interact with DDX5 in their subnetwork (Figures S1 B and C), which implied that the DDX5-centric subnetwork played a crucial role under AUF1 overexpression. DDX5 is a member of the DEAD-Box protein family, and it has been reported to be involved not only in various types of cancer, such as colorectal cancer, lung cancer, and hepatic cancer [30] but also in cases of viral infection. It showed a double-edged-sword effect on viral replication. For respiratory syndrome virus (PRRSV), previous data showed that DDX5 positively regulated the replication of PRRSV via its interaction with viral Nsp9 protein [31], while it negatively regulated the replication of two DNA viruses, hepatitis B virus (HBV), and myxoma virus (MYXV) [32]. In some contexts, knockdown of DDX5 and other DDXs, such as DDX3 and DDX52, could increase the replication of MYXV [33]. McFadden G et al. first clarified [33] that DDX5, DDX3, and some DDXs members have potential roles either in antiviral responses or regulation of innate immune responses. However, we detected the DDX5 expression level during CVB infection to explore the host response to viral infection. DDX5 was significantly increased under CVB infection (Figure 6(d)), while AUF1 significantly decreased due to cleavage. Compared with the siAUF1 treatment, there was still a slight increase in DDX5 (Figure 6(c)). In contrast, when AUF1 was overexpressed with a plasmid, DDX5 expression in host cells was significantly inhibited (Figure 6(b)). These results were sufficient to illustrate that AUF1 negatively regulated DDX5 at both the mRNA and protein levels. More biological effects of AUF1-regulated DDX5 on CVB should be further explored. Based on previous research, it most likely contributes to promoting CVB replication; however, the present study provides new insight into the mechanism of viral-induced pathogenetic injury to the host or the pathway that could be utilized by the host as an antiviral strategy.

## Data Availability

The mass spectrometry proteomics data have been deposited to the ProteomeXchange Consortium via the PRIDE 24 partner repository with the dataset identifier PXD021094.

## Supplementary Materials

Supplementary Materials Supplementary Figure S1: GO-BP in downregulated genes. Supplementary Figure S2: Subnetworks of other hubs. Supplementary Table 1: The principle KEGG enrichment divided into increased and decreased clusters. Supplementary file: The BP in GO analysis was performed on cluster 1, and detailed data are supplied.
