# Supplementary material for: Cleavage of AUF1 by Coxsackievirus B Affects DDX5 Regulatory on Viral Replication through iTRAQ Proteomics Analysis
Source: Biomed Res Int. 2022 Oct 6;2022:8610467. doi: 10.1155/2022/8610467 (PMC9560859; doi:10.1155/2022/8610467)
Supplement: Supplementary Materials — Supplementary Figure S1: GO-BP in downregulated genes. Supplementary Figure S2: Subnetworks of other hubs. Supplementary Table 1: The principle KEGG enrichment divided into increased and decreased clusters. Supplementary file: The BP in GO analysis was performed on cluster 1, and detailed data are supplied. [file 8610467.f1.zip › Supplementary Table 1-heatmap-two group-kegg form.pdf]

Supplementary Table 1. The principle KEGG enrichment divided into increased and decreased clusters

Pathway ID	Pathway description	Observed gene No.	Matching proteins
hsa03040	Spliceosome	15	CDC5L, **DDX42**, hnRNPA3, hnRNPU, LSM3, PRPF31, PUF60, SF3A1, SF3A2, snRNP40, snRPA, snRPE, SNW1, U2AF2, WBP11
hsa03010	Ribosome	10	MRPL2, MRPL3, MRPL4, MRPS11, MRPS5, RPL10A, RPL12, RPL9, RPS27A, RPS28
hsa03010	Ribosome	23	RPL11, RPL14, RPL18, RPL18A, RPL22, RPL23A, RPL31, RPL32, RPL35, RPL36, RPL5, RPLP0, RPS11, RPS12, RPS13, RPS15, RPS19, RPS20, RPS24, RPS3A, RPS6, RPS7, RPS9
hsa03040	Spliceosome	6	BUD31, CHERP, **DDX5**, snRPC, snRPD3, SRSF3
hsa03013	RNA transport	5	EIF3B, EIF3F, EIF3G, EIF4A1, PABPC1
