# Supplementary figures and images for: Cleavage of AUF1 by Coxsackievirus B Affects DDX5 Regulatory on Viral Replication through iTRAQ Proteomics Analysis
Source: Biomed Res Int. 2022 Oct 6;2022:8610467. doi: 10.1155/2022/8610467 (PMC9560859; doi:10.1155/2022/8610467)

Top of GO BP Enrichment for down-regulated proteins

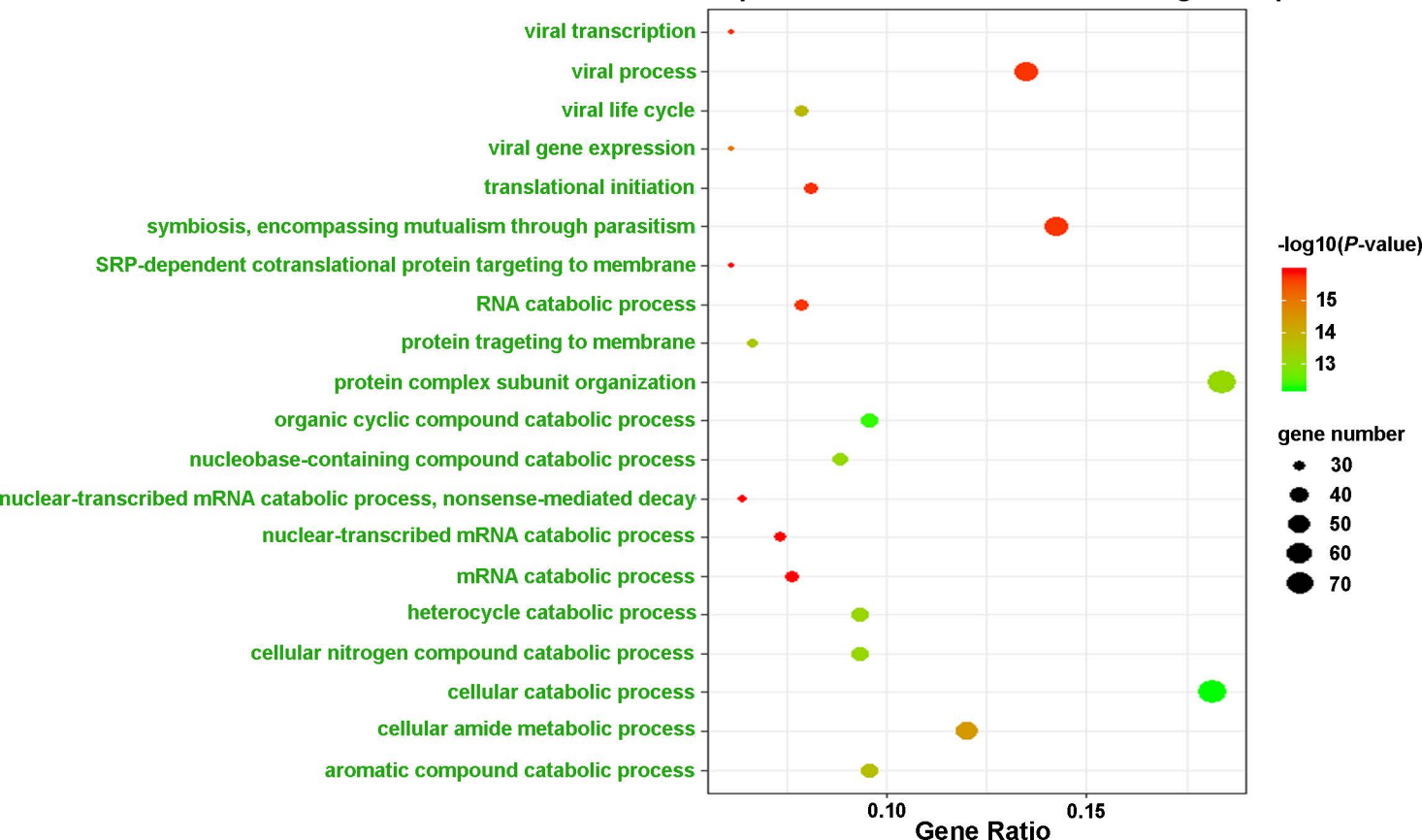

Supplement: Supplementary Materials — Supplementary Figure S1: GO-BP in downregulated genes. Supplementary Figure S2: Subnetworks of other hubs. Supplementary Table 1: The principle KEGG enrichment divided into increased and decreased clusters. Supplementary file: The BP in GO analysis was performed on cluster 1, and detailed data are supplied. [file 8610467.f1.zip › subSupplementary Figure 1-GO-BP in downregulated genes.pdf]

A

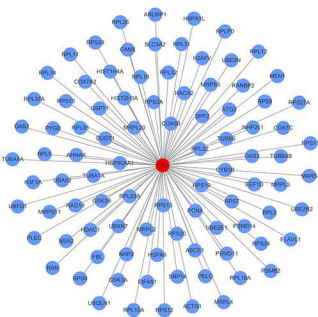

FAU

B

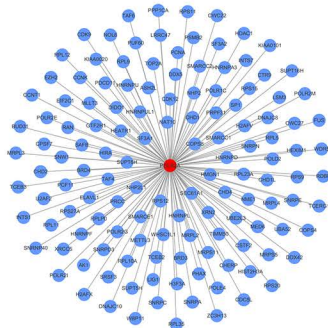

POLR2A

C

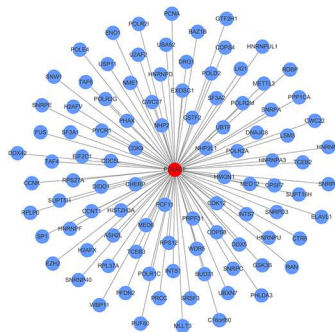

POLR2E

D

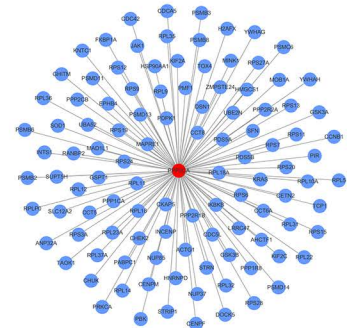

PPP2CA

E

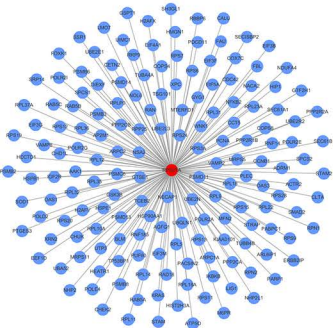

RPS27A

F

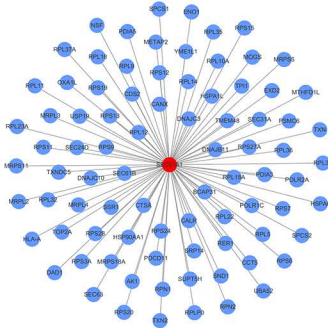

SEC61A1

G

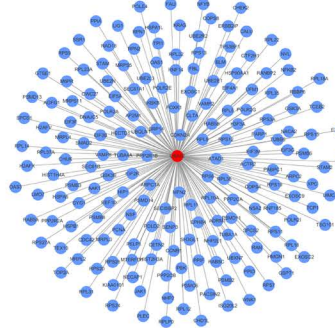

UBA52

H

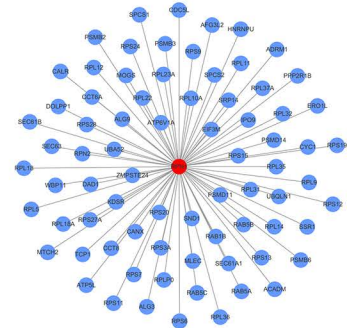

RPN1

Supplement: Supplementary Materials — Supplementary Figure S1: GO-BP in downregulated genes. Supplementary Figure S2: Subnetworks of other hubs. Supplementary Table 1: The principle KEGG enrichment divided into increased and decreased clusters. Supplementary file: The BP in GO analysis was performed on cluster 1, and detailed data are supplied. [file 8610467.f1.zip › subSupplementary Figure 2-subnetworks of other hubs.pdf]
